# Funding models and medical dominance in interdisciplinary primary care teams: qualitative evidence from three Canadian provinces

**DOI:** 10.1186/s12960-018-0299-3

**Published:** 2018-08-13

**Authors:** Wiesława Dominika Wranik, Susan Marie Haydt

**Affiliations:** 1 School of Public Administration, Faculty of Management, Dalhousie University, 6100 University Avenue, Halifax, Nova Scotia B3H 4R2 Canada; 2 Department of Community Health and Epidemiology, Faculty of Medicine, Dalhousie University, 6100 University Avenue, Halifax, Nova Scotia B3H 4R2 Canada; 3 Faculty of Management, Dalhousie University, 6100 University Avenue, Halifax, Nova Scotia B3H 4R2 Canada

## Abstract

**Background:**

Primary care in Canada is the first point of entry for patients needing specialized services, the fundamental source of care for those living with chronic illness, and the main supplier of preventive services. Increased pressures on the system lead to changes such as an increased reliance on interdisciplinary teams, which are advocated to have numerous advantages. The functioning of teams largely depends on inter-professional relationships that can be supported or strained by the financial arrangements within teams. We assess which types of financial environments perpetuate and which reduce the challenge of medical dominance.

**Methods:**

Using qualitative interview data from 19 interdisciplinary teams/networks in three Canadian provinces, as well as related policy documents, we develop a typology of financial environments along two dimensions, financial hierarchy and multiplicity of funding sources. A financial hierarchy is created when the incomes of some providers are a function of the incomes of other providers. A multiplicity of funding sources is created when team funding is provided by several funders and a team faces multiple lines of accountability.

**Results:**

We argue that medical dominance is perpetuated with higher degrees of financial hierarchy and higher degrees of multiplicity. We show that the financial environments created in the three provinces have not supported a reduction in medical dominance. The longstanding Community Health Centre model, however, displays the least financial hierarchy and the least multiplicity—an environment least fertile for medical dominance.

**Conclusions:**

The functioning of interdisciplinary primary care teams can be negatively affected by the unique positioning of the medical profession. The financial environment created for teams is an important consideration in policy development, as it plays an important role in establishing inter-professional relationships. Policies that reduce financial hierarchies and funding multiplicities are optimal in this regard.

**Electronic supplementary material:**

The online version of this article (10.1186/s12960-018-0299-3) contains supplementary material, which is available to authorized users.

## Background

For more than four decades, interdisciplinarity and team work in primary care (PC) have been hailed as key to improving health care systems. Interdisciplinary Primary Care (IDPC) teams are considered a potential solution to system problems such as a growing patient population and shortages of trained personnel [1], and advantageous with respect to health outcomes, clinical performance, care quality and chronic disease management [2–14]. IDPC teams may be preferred by patients [6, 7, 9, 15], and by providers [6–8, 11, 16, 17]. Lastly, some suggest improvements in system level outcomes, such as increased efficiency and reduced fragmentation [6, 9, 11, 17–20].

The literature focuses on the influence of various organizational factors on the functioning of IDPC teams. Facilitators of team functioning described in the literature include supportive, clear and transparent processes, institutional reinforcements, and a “sense of togetherness” [7, 8, 11, 12, 16, 17, 21–27]. Barriers to team functioning include insufficient education and training, the mismanagement of resources and team diversity and miscommunication [12, 22–24, 26, 28–33].

However, the literature is mostly silent on the description or effects of financing models for IDPC teams, aside from indicating that these are important [34–36]. The design of funding and remuneration models remains understudied in terms of options and impact on team functioning. Financing is considered important, with little analysis or discussion of optimal methods [37]. There is a lack of descriptive, comparative or evaluative studies of various approaches to the funding of teams and remuneration of providers within the PC context. The few studies we found focus on health care outcomes under three different care models with varying remuneration methods for physicians [38, 39], with two drawing a distinction between practice and provider level financial incentives, but not the interaction between them [18, 40]. A number of studies focus on the remuneration of individual providers (examples of systematic reviews include [41–44]), but this is generally not addressed at the team level. Some observe that the variety of remuneration methods within teams stand in the way of effective team practice [45]. Few studies incorporate a discussion about the remuneration of non-physician providers [46, 47]. In the majority of literature, the issues of interplay between team funding and provider remuneration, and between remuneration of physician and non-physician providers are ignored.

Primary care reform in Canada provides an interesting context to study the effects of reforms to financial models, given the country’s federalist system. PC reform in Canada has been ongoing for 16 years, with a large system-wide impetus toward IDPC teams in 2000 and 2006, when the Federal government provided support to its provinces and territories to redesign the delivery of PC through the Primary Care Transition Fund [48]. Further, the 2003 Health Accord identified “… access to an appropriate health care provider, 24 hours a day, 7 days a week…” as the ultimate goal of PC reform and consequently recognized the need to support “… multi-disciplinary primary health care organizations or teams” [49].

Given that the provinces and territories are in charge of deciding how federal funds are spent in their respective healthcare systems, PC reform was operationalized differently across regions in how IDPC teams were designed and implemented [50, 51]. Some provinces (e.g. Ontario, Alberta, Quebec and potentially Newfoundland) introduced system-wide policy changes to PC [52, 53], an approach we refer to as top-down overhaul. Other provinces (e.g. Manitoba, Saskatchewan) introduced quality improvement initiatives within traditional delivery models [53], an approach we refer to as top-down incremental. A third group (e.g. Nova Scotia, to some extent British Columbia) introduced incremental policy in response to existing changes in front-line delivery—what we refer to as a bottom-up incremental approach.

Yet despite differences in the paths of change to the organization, funding and delivery of PC, Canadian scholars have noted that some aspects, such as the persistence of fee-for-service payment for physicians [54], and physicians as de facto leaders of IDPC teams, remain very strong [55] and may act as barriers to IDPC teams [55]. These are believed to be structural remnants of medical dominance, which itself has been characterized as a barrier to the functioning of IDPC teams. By triggering the perception of threat to professional identities, it can become a cause for conflict and dysfunction in interdisciplinary teams [56]. Reduction in medical dominance has been argued to increase accountability and potentially support patient empowerment [57].

Medical dominance refers to the medical profession’s control over the content, terms and conditions of its own work (autonomy), control over other health occupations and the health division of labour (authority), control over clients and control over the broader context of health care (sovereignty) [58–62]. Medical dominance in Canada became firmly established throughout the twentieth century [63] and afforded physicians the power to assert influence on the administration of the health care system both actively through negotiation and passively through the centrality of their role.

While medical dominance has declined overall compared to the 1960s, the concept remains important internationally and particularly to studies of interdisciplinary practice [56]. While the dominance of the medical profession is challenged through various professional, legislative and cultural changes [64], it continues to act as a barrier to the professionalization and gaining of autonomy of other professions, such as nursing in Italy [65] or clinical pharmacy in Nigeria [66]. Stronger examples of conflict caused by medical dominance can be found in Nigeria in the form of strikes and other service disruptions [67].

The goals of our study are to characterize the implications that financial arrangements have on the balance of power in teams and whether financial models were perceived to influence the presence of professional hierarchies. First, we develop a general typology of financial arrangements for IDPC teams and identify the potential implications of each type on professional hierarchies. Second, we apply the typology to three lesser studied Canadian provinces, each an exemplar of a particular policy approach to recent PC reforms: Alberta is an example of the top-down overhaul approach, Manitoba is an example of the top-down incremental approach and Nova Scotia is an example of the bottom-up incremental approach. We compare these recent approaches to IDPC teams to the well-established Community Health Centre (CHC) model. We use the typology as a framework to assess which types of financial environments perpetuate and which reduce the challenge of medical dominance.

## Methods

We use data from a related qualitative study that describes financial incentives for collaboration within IDPC teams, as well as implementation and other emergent issues associated with the three approaches taken by various provinces [68]. In this paper, we conduct a re-analysis of original interview data and synthesized policy documents. Detailed information about the research team, data collection and analysis are provided in the checklist of consolidated criteria for reporting qualitative research (COREQ) in Additional file 1.

The original data set consisted of (i) policy documents describing financial/remuneration models in IDPC teams across Canada, (ii) semi-structured interviews to discuss the effects of these models on interdisciplinarity and collaboration and (iii) a research roundtable to discuss interim results and policy implementation issues. A detailed description of data collection can be found in Wranik et al. [68].

Policy documents were reviewed in several stages. Prior to the onset of the study, documents were reviewed in support of a related policy consultancy in 2012 and a subsequent development of an academic study protocol. Documents were searched on the websites of provincial ministries of health and regional health authorities. Searches were for IDPC, retrieved websites/documents were scanned for descriptions of financial arrangements and finally those containing descriptions of financial arrangements were included in final analysis. The aim of the document review was to describe the funding and remuneration models used in the three provinces. Document searches were updated between October and December 2013 at the onset of the study and supplemented with documents provided by policy decision-makers who participated in the original study as co-investigators.

Semi-structured qualitative interviews (*n* = 19) were conducted between January and May 2014. Purposive sampling was used to recruit leaders overseeing IDPC teams, such as executive directors, directors and/or managers (titles varied by province). In Nova Scotia, respondents were at the health authority level and oversaw more than one clinic. In Alberta, we interviewed executive directors of Primary Care Networks (PCNs) of varying sizes, most with multiple clinics, including one or more sites. In Manitoba, we interviewed primarily managers of individual clinics. Potential respondents were identified by the Department of Health and Wellness in Nova Scotia, by the Ministry of Health in Manitoba and by an online search for contacts of PCNs in Alberta.

A research roundtable with policy decision-makers from the three provinces was held in October 2014 in Halifax, Nova Scotia. The roundtable data are not a focus of this paper, except for the validation of proposed typologies that was provided through the discussions with decision-makers [69].

The study protocol was approved by the research ethics committees at nine district health authorities in Nova Scotia (since amalgamated into one), at the University of Manitoba and at Alberta Health. Interview respondents gave consent for the description of financial models and analysis of implications for a variety of health care goals.

The typology of financial arrangements and inter-professional relations serves as a framework for the discussion of the interplay between them [70]. During the original study, we developed several conceptual models of financial arrangements on the basis of the literature, documents and interviews and subsequently reviewed and revised them with study participants during the research roundtable, as well as policymaker co-investigators. For this paper, we focus on the version most appropriate for our study objective. It is important to note that interview respondents did not speak directly to the typology, which was developed later, and as such, results below are a post hoc interpretation of interview data within this framework.

Interview transcriptions were re-analysed by both authors using thematic content analysis as the primary analytical process. The authors initially coded the transcriptions independently and periodically conferred to calibrate results and interpret the themes with reference to the typology and the leading research question.

## Results

The 19 respondents represented six PCNs in Alberta (184 clinics), eight individual clinics in Manitoba and five district health authorities in Nova Scotia (23 clinics). They described the situations of approximately 735 medical doctors, 228 nurses and 145 other health care providers across three provinces. Further details breaking down the composition of each team and their goals are available in Additional file 2 and described in Wranik et al. [68].

### Conceptualizing financial models

**Fig. 1:**
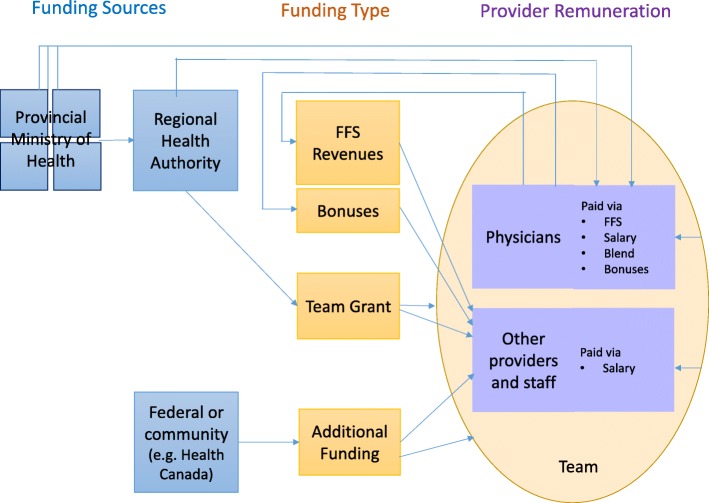
Multiplicity of funding flows and lines of accountability

The typology is constructed along two dimensions: (i) the degree of multiplicity of funding sources and (ii) the degree of financial hierarchy between providers. The multiplicity of funding sources and lines of accountability refers to the fact that some teams rely on many varied sources of funding, some of which are earmarked for specific services or specific populations. This introduces multiple and potentially conflicting lines of accountability. Multiplicity of funding is further unpacked with the use of a flow diagram (Fig. 1). The financial hierarchy between providers refers to the extent to which the income of one provider in the team depends on the activities of another provider. For example, in a traditional fee-for-services setting, the activities of the physician determine the funding available to pay other providers. This feature is retained, although covert, in some of the newer financial arrangements explored below. The financial hierarchy is explored in greater detail elsewhere [68].

**Fig. 2:**
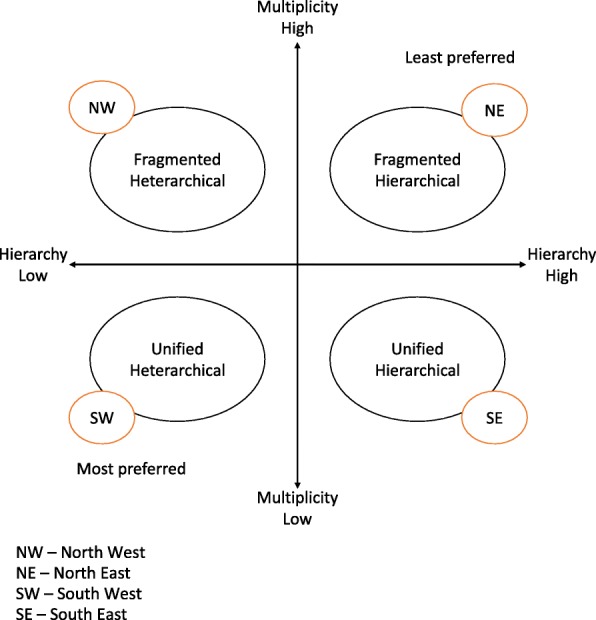
Typology of financial environments

The Provincial/Territorial Ministries of Health often use multiple budget envelopes to fund PC and is therefore shown as one set of several funding sources. Funding can flow directly to providers, or it can be pooled in part or in whole by an intermediary entity (e.g. PC network, PC team), thereby reducing multiplicity. The degree of multiplicity increases with the number of funders, as do the associated number of lines of accountability, and the extent of conflict between multiple sets of priorities. Greater multiplicity reduces the opportunity to dismantle medical dominance due to the continued positioning of the medical profession as unique in terms of funding source and accountability. For purposes of the typology in Fig. 2, we identify two types along the multiplicity scale—unified (low multiplicity) and fragmented (high multiplicity).

Financial hierarchy between providers, the second dimension of the typology, is greatest, when one provider’s activities generate the revenues that are the source of another provider’s income. This is the traditional model, where a physician receives fee-for-service compensation and using this money pays a nurse. Hierarchy is least when all providers are paid by a third party, and the income of one is not linked to the activities of the other. We postulate here that financial heterarchy is a necessary, although not sufficient condition to the dismantling of medical dominance.

Figure 2 captures four types, four combinations between high and low multiplicity, and high and low hierarchy. The Fragmented Hierarchical type in the north-east quadrant creates financial conditions that most perpetuate the medical dominance legacy, whereas the Unified Heterarchical type in the south-west quadrant is most likely to challenge the legacy. Conceptually then, policies that result in movements toward the south and west are preferred.

### Perceived implications of financial models on medical dominance

Traditionally, PC physicians in Canada operated as sole providers. In this arrangement, interdisciplinarity could be added by hiring a nurse. This financial model can be placed in the south-east corner of the typology; it perpetuates the legacy of physician dominance through a financial hierarchy. Whereas a preferred policy path would be to move toward the south-west, where financial hierarchy is lower and financial models are unified, our results suggest that newer financial models have introduced greater multiplicity and have therefore moved north. Several sites interviewed in Manitoba followed the CHC model that has existed across Canada since the 1970s. Their positioning in the typology is closest to the preferred south-west quadrant.

The top-down incremental approach in Manitoba created relatively little movement along the hierarchy scale. Newer arrangements (Physician Integrated Network, myHealth Teams) continued to rely on the fee-for-service system to remunerate physicians, but offered bonus payments to encourage interdisciplinarity within this system (minimal movement west). The core structure of physician-generated revenues being the source of income for other providers has been retained, while some staff within clinics have been employed by the regional health authority. Many were drawing on secondary funding sources though Health Canada, Manitoba Health or the Addictions Foundation of Canada, creating multiplicity (movement north). Where followed, the CHC structure allowed for a pooling of multiple sources and therefore a less fragmented model.

“Again, most of the other positions we have are funded by the clinic itself, which is funded through fee-for-service billings through physicians and then the physicians turning around and hiring these positions” (MB8)

The top-down overhaul approach in Alberta created a relatively greater stride away from a financial hierarchy in comparison to Manitoba’s models, but the funding for PCNs remained closely tied to the activities of physicians. Funding for PCNs came in the form of a capitation payment per patient rostered to participating physicians. The financial dependence of health providers’ salaries on activities of physicians is indirect, but remains. Alberta’s PCNs also draw on additional funding sources; in contrast with Manitoba’s myHealth Teams or Physician Integrated Network clinics, however, they act as an intermediary between funders and providers thereby mitigating some challenges of multiplicity. Alberta appears to have moved toward the north-west, as well, but farther west and not as far north as Manitoba.

“ […] from [our] patient panel, we get a certain amount of money per patient attached to the PCN, $62 per patient, and then from there we use those funds appropriately. And then that’s a discussion between senior management and the physician boards to see how everything needs to be split up and what services need and that kind of stuff.” (AB1)

The funding multiplicity can be mitigated through the pooling of funding sources by an entity, in Alberta’s case the PCN. The pooling function can act to reduce the uniqueness of the medical professional’s roles by consolidating the lines of accountability into one.

“On the occasion where we have physicians coming […] to see patients at the PCN’s office, we take them off of fee-for-service and we put them on what sessional rate to try to match closer to what the rest of the staff are doing.” (AB6)

The bottom-up incremental approach in Nova Scotia appears to move the farthest east in terms of creating a heterarchical financial environment. All providers, including physicians in PC Teams receive salaries through the Ministry of Health or Regional Health Authority. Nova Scotia’s financial environment, however, continues to suffer from a subtle, yet impactful multiplicity—physicians are paid from a separate budget envelope within the Ministry of Health. While their activities no longer determine the amount of funding available for other providers, physicians continue to assume a unique position in the team (autonomy).

“We, [i.e. the PC team] have control over everybody [nurse practitioners, dietician, RN] except the physicians, who are managed by Physician Services… the fact that physicians are not in the same .. that they are funded by an outside body doesn’t necessarily make them accountable for the work they do.” (NS2)

Another respondent from Nova Scotia noted that while the entity manager controlled the hiring process with input from physicians, clinical decision-making remained firmly with physicians vis-à-vis other team members. In this case, physician control over the work of other health professions (authority) remains despite the change in financial remuneration.

“The physician would have the final say ... because technically in our set-up, the patients are [often] rostered to the physician. So the physician is ultimately responsible for overall care, and would be even responsible for other team members’ actions” (NS1)

The CHC model is closest to the south-west corner of the typology. The CHC acts as a separate entity, which pools funding from multiple sources, and disburses as salaries to all providers including physicians. The multiplicity of funding is mitigated through the CHC as an entity, as highlighted by the existence of a “team” in Fig. 1. As noted, the CHC model has existed since the 1970s, and we note that it had more success with creating a level playing field for all health professionals compared to more recent reforms.

Importantly, our data suggests that medical dominance continues to play in indirect ways in addition to the direct impact of financial incentives as discussed above. Most influential here is the centrality of the patient-physician relationship that is often maintained despite nominal commitments to shifting focus away from it. The centrality is preserved through the very understanding of how interdisciplinary team is defined, and also through matters corollary to financial arrangements, such as patient rostering requirements, and the funding of space and equipment. For example, one PCN in Alberta describes the team as:

“… we send [team members] out into physicians’ offices […] we call that the core team. […] It always includes a physician and the patient because we want to focus on that. We call it the Health Home but it’s also called a Medical Home. But then within that Home, we also try to give extra support by providing… a registered nurse, pharmacist, and...a mental health worker […] access to dieticians” (AB1).

This stands in contrast with the description provided by a CHC in Manitoba:

“So when I think about the team, I think about our patient or client in the centre. So the full team around them may be different for each client but members of the team are sort of divided into […] a clinical team, […] a health promotion and allied care team, and then […] our health education and prevention team who don't work directly with our clients very much one-on-one but may do more work with at-risk groups” (MB3)

As noted, the rostering of patients appears as an influential mediating factor between financial arrangements and professional hierarchy. For example, patients may be required to roster with a physician in their catchment area, or they are rostered with a physician in order to access services of a clinic. The implication is that the physician often controls how the patient subsequently interacts with other health providers in the team by controlling the division of labour and/or retaining control over clinical decisions.

“So currently […] the patients are attached to a physician in our practice. So they aren’t directly attached to another care provider. […] the physician engages the other care provider, depending on, you know, what kind of involvement this other care provider will have, depending on their scope of practice and what it is they're doing and what type of patient they're providing care with” (MB8)*.*

A second mediating factor is the question of negotiating funding for space and equipment. This issue is challenging across provinces and it is resolved in a relatively ad hoc manner at the team level. Many arrangements in this regard perpetuate the dominant position of the medical doctor.

“…sometimes within the physician's office, because they are running a business as well, one of the things is they provide us [members of the team] some space to work in… sometimes the clinic only has space for one member on a certain day kind of thing. So when I say there's 3 members in the office, it won't necessarily be 3 people all together at once. It might be the nurse on a Tuesday, the BHC on a Wednesday and a pharmacist on a Thursday (AB1).”

“Even though the PCN will pay the salary and put in a highly skilled person into an office, there's pushback [by physicians] on that because of the risk of not being able to charge for that patient appointment kind of thing… So if you're strictly fee-for-service, and then we're trying to put a nurse in in one of your exam rooms, so now you're down to one exam room, you know, you've just cut your patient load for that day in half kind of thing”(AB4)*.*

To sum up, each of the three avenues have moved the financial models away from a financial hierarchy to varying degrees, while simultaneously adding undesirable financial multiplicity. Corollary aspects that perpetuate professional hierarchies are the questions of how patient rosters are established, specifically to which provider patients are assigned, and how funding for space and equipment is secured.

## Discussion

Our findings are aligned with previous studies of the state of medical dominance in Canada and elsewhere, although we contribute to the literature by exploring the issue of medical dominance in the context of financial arrangements in IDPC teams specifically. The phenomenon persists in Canada, despite various degrees of change to PC delivery Canada [55, 58, 60]. It is considered a hindrance to the development of other health care professions [64, 65], can lead to conflict between providers [67] and is therefore undesirable when the goal is to support interdisciplinary PC.

Policies aiming to improve PC delivery should focus on creating financial environments that are least fertile to the challenge of medical dominance. As discussed in the literature, medical dominance is seen as an obstacle to team functionality. While the absence of the authority of one person within a team to coordinate and make final decisions could lead to indecision or chaos, this is not documented, nor is there any argument to suggest that this person ought to be a physician. On the contrary, we suggest that the existing CHC model outperforms the newer types of models. This is consistent with recent findings of superiority of the CHC in chronic disease management [10, 71], access, prevention, promotion, patient and family centeredness, overall patient experience and other primary care goals [71] .

The limitations of our study are that our respondents consisted of individuals in charge of teams, rather than front-line providers, and therefore their perceptions of team interactions were from a distance. Several respondents were motivated to speak positively about “their” team, particularly when they had been personally involved in the design or implementation of the team. Furthermore, a more complete picture could be drawn with data from more than three provinces.

The development of a typology is a first step to generalizable evaluative studies that can speak to the successes, failure, strengths and weaknesses of particular types of funding and remuneration approaches. We know from other studies [72] that our extant knowledge about the impact of financial arrangements on processes, health care outputs and/or health outcomes in PC is very limited. Questions of how well the various types of financial arrangements perform in terms of improving service delivery or improving patient health would best be addressed via quantitative analysis in the future.

## Conclusions

The financial environment is one of the discretionary policy levers available to decision-makers who wish to affect the functioning of PC delivery. Our division of the financial environment into two layers, funding of care and remuneration of individual providers, allows us to describe how these two dimensions affect hierarchical relationships between providers. This issue is particularly important to consider in the interdisciplinary PC setting.

The financial environment can be visualized as a breeding ground for inter-professional relationships, one that can be more or less fertile to the negative effects of medical dominance on such relationships. This with the understanding that the financial environment is but one factor that influences relationships between professions. We identify the fragmented and financially hierarchical model as the most fertile ground to medical dominance. We also show that newer financial environments used in three Canadian provinces continue to support medical dominance by continuing to place physicians in a unique financial position.

Physicians appear to have retained many of the features of medical dominance. Even in models where physicians and all other providers receive a salary, but the source of funding differs, physicians retain autonomy because they are not financially accountable to their team manager. We also showed that situations where physicians’ activities remain tied to the funding for the team and/or remuneration of other providers, physicians retain authority over the work of other team members. Furthermore, the monopoly over medical knowledge (sovereignty), while arguably depleted through other efforts not within the scope of this study, is not challenged by the financial hierarchy present in most models we study.

## Additional files

Additional file 1: COREQ—consolidated criteria for reporting qualitative research—checklist. (DOCX 19 kb)

Additional file 2: Profiles of networks/teams included in the study. (DOCX 25 kb)

## Abbreviations

CHC: Community Health Centre
COREQ: Consolidated criteria for reporting qualitative research
FFS: Fee-for-service
IDPC: Interdisciplinary Primary Care
NE: North-east
NW: North-west
PC: Primary care
PCN: Primary Care Network
SE: South-east
SW: South-west
