# Supplementary material for: Funding models and medical dominance in interdisciplinary primary care teams: qualitative evidence from three Canadian provinces
Source: Hum Resour Health. 2018 Aug 13;16:38. doi: 10.1186/s12960-018-0299-3 (PMC6090795; doi:10.1186/s12960-018-0299-3)
Supplement: Supplementary file 1 — COREQ—consolidated criteria for reporting qualitative research—checklist. (DOCX 19 kb) [file 12960_2018_299_MOESM1_ESM.docx]

**Additional file 1 - COREQ – Consolidated criteria for reporting qualitative research - Checklist**

Tong A. Sainsbury P. Craig J. Consolidated criteria for reporting qualitative research (COREQ): a 32-item checklist for interviews and focus groups. International Journal for Quality in Health Care. 19(6):349-357

| **Item** | **Guide questions/ description** | **Response** |
| --- | --- | --- |
| ***Domain 1: Research team and reflexivity*** | | |
| *Personal Characteristics* | | |
| 1. Interviewer/ facilitator | Which author(s) conducted interviews or focus groups? | DW (and research assistants= RAs) |
| 2. Credentials | What were the researchers credentials? E.g. PhD, MD | DW hold a PhD, SH holds a PhD, RAs had a undergraduate degree, one a graduate degree |
| 3. Occupation | What was their occupation at the time of study? | DW is an Associate Professor, SH is a Research Facilitator, RAs were graduate students |
| 4. Gender | Was the researcher male or female? | DW is female, SH is female, one RA is male |
| 5. Experience and training | What experience or training did the researcher have? | DW is an experienced interviewer in professional settings, SH is an expert in qualitative methods for data analysis, RAs had graduate level training, and were additionally trained by DW |
| *Relationship with Participants* | | |
| 6. Relationship established | Was a relationship established prior to study commencement? | Some respondents were previously professionally associated as co-authors in an associated study. |
| 7. Participant knowledge of the interviewer | What did the participants know about the researcher? E.g. personal goals, reasons for doing the research | Participants were informed about the reasons for the research via the invitation to participate, and the consent form. In addition, the study program has a public website. |
| 8. Interviewer characteristics | What characteristics were reported about the interviewer/facilitator? Bias, assumptions, reasons and interests in the research topic? | Researcher profiles are available on the study website, where professional paradigms are described (e.g. health economist, sociologist, epidemiologist, decision maker etc.) |
| ***Domain 2: Study design*** | | |
| *Theoretical framework* | | |
| 9. Methodological orientation and theory | What methodological orientation was stated to underpin the study? E.g. grounded theory, discourse analysis, ethnography, phenomenology, content analysis | The study framework was a policy analysis. Qualitative thematic analysis was applied to interviews with the goal to isolate policy component parts and analyse the relationships between them. |
| *Participant Selection* | | |
| 10.Sampling | How were participants selected? E.g. purposive, convenience, consecutive, snowball | The sample was purposive in that we approached participants in the positions of manager or director of IDPC teams. |
| 11. Method of approach | How were participants approached? e.g. face-to-face, telephone, email, mail | Participants were approached via email or telephone. Participants in Alberta were approached via email on a “cold-call” basis, participants in Manitoba and Nova Scotia were approached via email, but knew some of the authors of a related study professionally. |
| 12. Sample size | How many participants were in the study? | 19 managers/ directors were interviewed. 14 individuals participated in the roundtable, of which 5 were also interview respondents, 4 were also study co-authors in a related study, and 5 were new to the study. |
| 13. Non-participation | Now many participants refused to participate or dropped out? Reasons? | One in Nova Scotia, three in Manitoba were unable to commit the time to the study. In Alberta, 42 primary care networks were invited via email, and follow up phone call. |
| *Setting* | | |
| 14. Setting of data collection | Where was the data collected? E.g. home, clinic, workplace | Interview data were collected via telephone, respondents were not asked about their physical location. |
| 15. Presence of non-participants | Was anyone else present besides the participants and researchers? | During telephone interviews, RAs were accompanied by DW for the first three interviews, then were unaccompanied. Respondents were not asked about the presence of others. |
| 16.Description of sample | What are the important characteristics in the sample? E.g. demographic data, date | Participants were selected, if their professional role was to oversee IDPC team(s) either as managers, or directors. Participants for the roundtable also included policy decision makers. |
| *Data collection* | | |
| 17. Interview guide | Were questions, prompts, guides provided by the authors? Was it pilot tested? | Initial interview questions were developed by DW and discussed by the study team, and validated by co-authors of a related article, who are policy decision makers in primary care. The questions were also reviewed by appropriate ethics review boards. |
| 18. Repeat interviews | Were repeat interviews carried out? If yes, how many? | On six occasions, follow-up contact was established because initial responses were incomplete or not clear. |
| 19. Audio/ visual recording | Did the research use audio or visual recording to collect data? | All interviews were audio recorded and transcribed. |
| 20. Field notes | Were fields notes made during and/or after the interview or focus group? | Interviews were recorded and transcribed. Some notes were taken during interviews. |
| 21. Duration | What was the duration of the interviews or focus groups? | Each interview lasted approximately one hour. |
| 22. Data saturation | Was data saturation discussed? | Interviews in Alberta revealed that the funding/ remuneration approach was uniform across the Province, and the challenges experienced were similar, though varied by size and location of the PCN network. Interviews in Manitoba and Nova scotia revealed a diversity of financing approaches, but relatively similar challenges. It is the conclusion that additional interviews in the three selected provinces would not bring to light additional themes. |
| 23. Transcripts returned | Were transcripts returned to participants for comment and/or correction? | Interview transcripts were verified in case of lack of clarity (addressed under 18. Repeat interviews). Several interviewees provided written response to some of the questions. Transcripts were not provided to respondents in their final versions, and no such promise was made. |
| ***Domain 3: Analysis and findings*** | | |
| *Data analysis* | | |
| 24. Number of data coders | How many data coders coded the data? | DW and SH were responsible for coding, with assistance from RAs. DW and SH discussed themes. |
| 25. Description of coding tree | Did authors provide a description of the coding tree? | An emergent textual analysis of interview transcriptions was used. |
| 26. Deviation of themes | Were themes identified in advance or derived from the data | Evaluative components of the interviews were thematically coded ex post. |
| 27. Software | What software, if applicable, was used to manage the data? | Only Word and Excel were used. |
| 28. Participant checking | Did participants provide feedback on the findings? | A subset of participants was given the opportunity for feedback. A working paper report is publicly available online. |
| *Reporting* |  |  |
| 29. Quotations presented | Were participant quotations presented to illustrate themes/ findings? Was each quotation identified? E.g. participant number | Quotations are provided but are not attributed to particular respondents / type of respondents. |
| 30. Data and findings consistent | Was there consistency between the data presented and the findings? | Yes. |
| 31. Clarity of major themes | Were major themes clearly presented in the findings? | Major themes are presented and captured in the form of a conceptual framework. The framework is applied to the analysis of the particular issue of collaboration. Four major implementation issues are discussed. |
| 32. Clarity of minor themes | Is there a description of diverse cases or discussion of minor themes? | There were no deviating cases/ and in addition, respondents were informed that only aggregate results would be presented. Minor themes were not discussed to keep focus. |
