# Supplementary material for: Funding models and medical dominance in interdisciplinary primary care teams: qualitative evidence from three Canadian provinces
Source: Hum Resour Health. 2018 Aug 13;16:38. doi: 10.1186/s12960-018-0299-3 (PMC6090795; doi:10.1186/s12960-018-0299-3)
Supplement: Supplementary file 2 — Profiles of networks/teams included in the study. (DOCX 25 kb) [file 12960_2018_299_MOESM2_ESM.docx]

***Additional file 2 – Profiles of networks/teams included in the study***

| *ID* | *Number of clinics* | *Number of patients* | ***Physicians*** | | | ***Nurses/ Nurse Practitioners*** | | | ***Other care providers*** | | | ***Non-provider staff*** | | |
| --- | --- | --- | --- | --- | --- | --- | --- | --- | --- | --- | --- | --- | --- | --- |
|  |  |  | *Number* | *Remuneration* | *Source* | *Number* | *Remuneration* | *Source* | *Number* | *Remuneration* | *Source* | *Number* | *Remuneration* | *Source* |
| *AB1* | *86* | *387,000* | *385* | *FFS* | *DH* | *55* | *salary* | *PCN* | *39* | *salary* | *PCN* |  | *salary* | *PCN* |
| *AB2* | *37* | *100,000* | *82* | *FFS* | *DH* | *51* | *salary* | *PCN* | *13* | *salary* | *PCN* |  | *salary* | *PCN* |
| *AB3* | *12* | *23,000* | *21* | *FFS* | *DH* | *10* | *salary* | *PCN* | *2* | *salary* | *PCN* |  | *salary* | *PCN* |
| *AB4* | *19* | *120,000* | *82* | *hourly* | *DH* | *12* | *salary* | *PCN* | *13* | *salary* | *PCN* |  | *salary* | *PCN* |
| *AB5* | *1* | *20,000* | *2* | *FFS* | *DH* | *3* | *salary* | *PCN* | *1* | *salary* | *PCN* |  | *salary* | *PCN* |
| *AB6* | *29* | *87,000* | *65* | *FFS* | *DH* | *27* | *salary* | *PCN* | *9* | *salary* | *PCN* |  | *salary* | *PCN* |
| *MB1* | *1* | *-* | *4* | *FFS/salary* | *DH/HA* | *8* | *salary* | *HA* | *43* | *salary* | *DH/HA* |  | *salary* | *DH/HA* |
| *MB2* | *1* | *850* | *3* | *FFS/salary* | *DH/HA* | *6* | *salary* | *HA* | *4* | *salary* | *HA* |  | *salary* | *HA* |
| *MB3* | *1* | *-* | *3* | *FFS/salary* | *DH/HA* | *4* | *salary* | *HA* | *ns* | *salary* | *HA* |  | *salary* | *HA* |
| *MB4* | *1* | *-* | *1* | *FFS* | *DH* | *4* | *salary* | *HA* | *2* | *salary* | *HA/other* |  | *salary* | *HA* |
| *MB5* | *1* | *-* | *1* | *salary* | *HA* | *9* | *salary* | *HA* | *4* | *salary* | *HA* |  | *salary* | *HA* |
| *MB6* | *1* | *-* | *6* | *salary* | *HA* | *5* | *salary* | *HA* | *3* | *salary* | *HA* |  | *salary* | *HA* |
| *MB7* | *1* | *-* | *28* | *FFS/salary* | *DH/HA* | *9* | *salary* | *clinic (FFS)* | *2* | *hourly* | *patients* | *ns* | *ns* | *ns* |
| *MB8* | *1* | *30,000* | *19* | *FFS* | *DH* | *1* | *salary* | *clinic (FFS)* | *1* | *salary* | *HA* |  | *salary* | *clinic (FFS)* |
| *NS1* | *8* | *13,000* | *ns* | *salary/FFS* | *APP/ DH* | *ns* | *salary* | *HA + other* | *ns* | *salary* |  | *ns* | *salary* | *HA* |
| *NS2* | *1* | *ns* | *1* | *salary* | *APP (DH)* | *2* | *salary* | *HA* | *3* | *salary* | *HA* | *ns* | *salary* | *HA* |
| *NS3* | *3* | *11,000* | *~ 6* | *salary* | *APP (DH)* | *~ 3* | *salary* | *HA* | *-* | *-* | *-* | *ns* | *salary* | *HA* |
| *NS4* | *4* | *14,000* | *13* | *salary* | *APP (DH)* | *10* | *salary* | *HA* | *6* | *salary* | *HA* | *ns* | *salary* | *HA* |
| *NS5* | *7* | *10,000* | *12* | *salary* | *APP (DH)* | *8* | *salary* | *HA* | *ns* | *salary* |  | *ns* | *salary* | *HA* |
| ** Respondent ID indicates the province – AB is Alberta, MB is Manitoba, NS is Nova Scotia.*  ** Respondents in MB indicated that a panel size was not recorded. One clinic is a specialty clinic with 850 clients. One clinic estimated the number of potential patients in the geographical catchment area.*  **Funding sources are coded as: DH – Department of Health (names vary across provinces and over time); HA – Health Authority (names vary across provinces and over time); ARP – Alternative Payment Plan (includes any salary contract for physicians, often accompanied by shadow billing requirements); PCN – Primary Care Network (whole team or network grant);*  *ns – number not specified (e.g. some, several)* | | | | | | | | | | | | | | |

(Originally published in Wranik WD. Haydt SM. Katz A. Levy A. Korchagina M. Edwards J. Bower I. 2017. Funding and remuneration of interdisciplinary primary care teams in Canada: A conceptual framework and application)
